# Chronic diseases, chest computed tomography, and laboratory tests as predictors of severe respiratory failure and death in elderly Brazilian patients hospitalized with COVID-19: a prospective cohort study

**DOI:** 10.1186/s12877-022-02776-3

**Published:** 2022-02-17

**Authors:** Alberto Frisoli Junior, Elaine Azevedo, Angela Tavares Paes, Eliene Lima, João Carlos Campos Guerra, Sheila Jean Mc Neill Ingham

**Affiliations:** 1 Hospital Israelita Albert Einstein, Avenida Albert Einstein, 627. 1° Subsolo, Bloco B, Morumbi, São Paulo, SP Zip code : 05652-900 Brazil; 2 Elderly Vulnerability Disease Research Group Unit, Cardiology Division, Federal University of São Paulo, Rua Napoleão de Barros, 715 – Térreo- Vila Clementino, São Paulo, SP Zip code: 04024-002 Brazil; 3 Osteometabolic Diseases Unit - Hospital do Servidor Público Estadual de São Paulo Av. Ibirapuera, 981. Vila Clementino, São Paulo, SP Zip code: 04038 Brazil; 4 Statistics Department, Federal University of São Paulo, Rua Diogo de Faria, 1087. 4 andar, cj 408- Vila Clementino, São Paulo, SP Zip code: 04037003 Brazil

**Keywords:** COVID-19, Respiratory failure, Elderly, Mortality, Vitamin D, Anemia

## Abstract

**Background:**

The primary risk factors for severe respiratory failure and death in the elderly hospitalized with COVID-19 remain unclear.

**Objective:**

To determine the association of chronic diseases, chest computed tomography (CT), and laboratory tests with severe respiratory failure and mortality in older adults hospitalized with COVID-19.

**Method:**

This was a prospective cohort with 201 hospitalized older adults with COVID-19. Chronic diseases, chest CT, laboratory tests, and other data were collected within the first 48 h of hospitalization. Outcomes were progression to severe respiratory failure with the need of mechanical ventilation (SRF/MV) and death.

**Results:**

The mean age was 72.7 ± 9.2 years, and 63.2% were men. SRF/MV occurred in 16.9% (*p* < 0.001), and death occurred in 8%. In the adjusted regression analyses, lung involvement over 50% [odds ratio (OR): 3.09 (1.03–9.28; 0.043)], C-reactive protein (CRP) > 80 ng/mL [OR: 2.97 (0.99–8.93; 0.052)], Vitamin D < 40 ng/mL [OR: 6.41 (1.21–33.88; 0.029)], and hemoglobin < 12 g/mL [OR: 3.32 (1.20–9.20; 0.020)] were independent predictors for SFR/MV, while chronic atrial fibrillation [OR: 26.72 (3.87–184.11; 0.001)], cancer history [OR:8.32 (1.28–53.91; 0.026)] and IL-6 > 40 pg/mL [OR:10.01 (1.66–60.13; 0.012)] were independent predictors of death.

**Conclusion:**

In hospitalized older adults with COVID-19, tomographic pulmonary involvement > 50%, anemia, vitamin D below 40 ng/mL, and CRP above 80 mg/L were independent risk factors for progression to SRF/MV. The presence of chronic atrial fibrillation, previous cancer, IL-6 > 40 pg/mL, and anemia were independent predictors of death.

## Introduction

The COVID-19 pandemic has been characterized by severe impacts on the elderly population, who suffered high hospitalization rates, unfavorable clinical evolution, and substantial mortality [1]. In the United States, older adults accounted for 31% of cases with clinical manifestations, 45% of hospitalizations, and 53% of admissions to intensive care units (ICU). Approximately 80% of fatal cases occurred among people over 65 years old, 46% between 65 and 84 years old, and 34% among those over 85 years old [1].

Acute respiratory distress syndrome is the primary clinical manifestation of severe disease and the strongest predictor of mortality from COVID-19, especially when requiring mechanical ventilation [2]. Age above 65 years is an independent predictor for acute respiratory distress syndrome [3], and this could be caused by the dysfunction of the immune system associated with aging, with exacerbated inflammatory reactions and decreased anti-inflammatory responses (Inflammaging) [4]. Additionally, the elderly have a higher prevalence of chronic diseases, physical frailty, and malnutrition that stimulate the increase in inflammatory interleukins and hypercoagulation states [5, 6]. Laboratory values that can help diagnose these conditions are high levels of neutrophils, C-reactive protein (CRP), troponin, myoglobin, d-dimer, lactate dehydrogenase (LDH), and IL-6; these parameters and low lymphocyte counts predict progression to respiratory failure and death [7–10].

Nevertheless, the relationship of chronic diseases and imaging and laboratory parameters with the development of respiratory failure and mortality due to COVID-19 in the elderly remains poorly understood [6, 11, 12]. To better understand these associations, we determined the predictive value of chronic comorbidities, pulmonary involvement by chest CT, and laboratory tests at the time of hospitalization, with respiratory failure requiring mechanical ventilation and mortality in an elderly population hospitalized with SARS-Cov-2 infection.

## Methods

### Study design

This was a prospective cohort study of older adults with confirmed SARS-Cov-2 infection hospitalized for COVID-19.

### Study sample

**Fig. 1:**
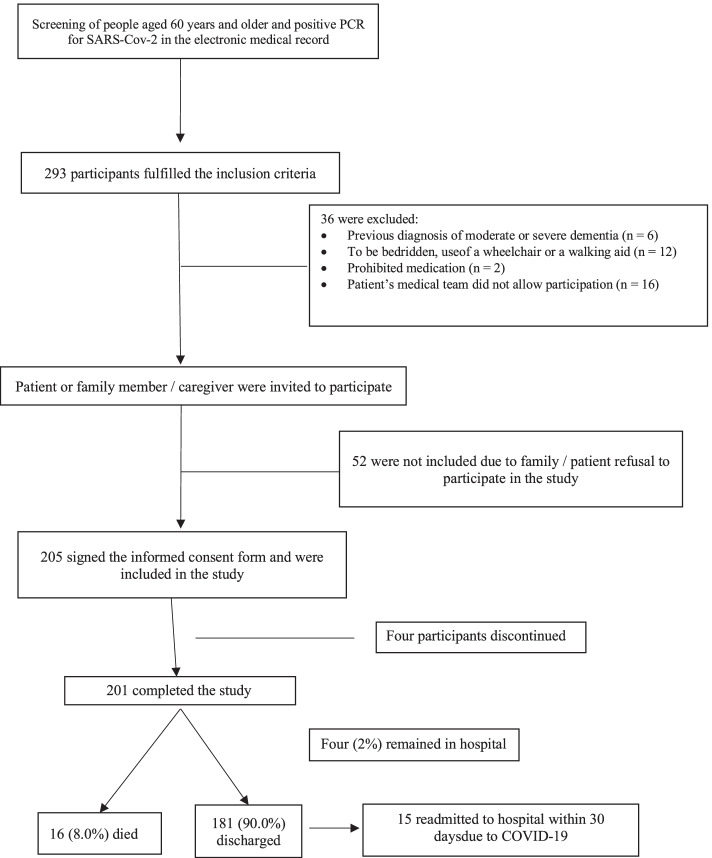
Flowchart of the study design

We considered 201 participants (Fig. 1). Inclusion criteria were age over 60 years and have a positive RT-PCR for SARS-CoV-2 at the time of hospitalization. Exclusion criteria were previously diagnosed moderate or severe dementia, use of prohibited medications (previous use of corticosteroids > 7.5 mg/day of methyl prednisolone for more than 3 months, use of immunosuppressants, chemotherapeutic agents, biological immunomodulators).

This study followed the relevant ethical guidelines and regulations as described in the Declaration of Helsinki and was approved by the Ethics Committee of the Hospital Israelita Albert Einstein and of Plataforma Brasil under the numbers: 4.082.096 e 4.186.636.

All participants, caregivers, or family members provided written or electronic consent (after a telephone conversation in the latter’s case). Participants were followed during their entire hospital stay and up to 30 days after hospital discharge. Four inpatients remained at the end of the study, all with more than 60 days in the hospital.

### Variables

Demographic data, ethnicity, health habits (smoking and drinking), education (years) and marital status, and variables of interest (described below) were evaluated. All information was collected by phone call or from the electronic medical record.

#### Concomitant medications

Information on chronic medications was obtained. During hospitalization, uses of corticosteroids (oral or intravenous), vitamin D, tocilizumab, oseltamivir, remdesivir, and convalescent plasma were evaluated.

#### Chronic diseases

Hypertension, diabetes mellitus, heart failure, previous myocardial infarction, atrial fibrillation, history of stroke, asthma, osteoporosis, osteoarthritis, previous bone fracture, Parkinson’s disease, Alzheimer’s disease, chronic kidney disease (CKD) (non-hemodialysis (HD)), end-stage renal disease (on HD), current and previous cancer, falls in the previous 6 months, and physical frailty. Chronic diseases were assessed with a family member or caregiver.

To assess the presence of physical frailty, the FRAIL questionnaire [13] was used as it does not involve any physical performance activities to avoid the risk of staff contamination and recognizing the limits of the participants’ clinical conditions. A final score of 3 or more was considered frail, while 1 or 2 indicated pre-frail, and zero indicated robust.

#### Laboratory tests

Complete blood count (hemoglobin (Hb), total leucocytes (TLeuc), total lymphocytes (TLymp), 25-hydroxyvitamin D (25(OH)D), alanine transaminase (ALT), aspartate aminotransferase (AST), CRP, interleukin 6 (IL-6), D-dimer, creatinine (Creat), myoglobin (Myo) were collected during the first 48 h of hospitalization. Laboratory tests were assessed as categorical variables in the form of quartiles.

The following quartiles were used in the analyses of the association with clinical outcomes:

- Lower quartiles:
  - Hemoglobin (< 11.7 g/mL, approximated to 12 g/ml, was defined as anemia)
  - Total lymphocytes (< 597/μL, approximated to 600/μL)
- Higher quartiles:
  - D-dimer (> 1046 ng/mL, approximated to 1000 ng/mL)
  - CRP (> 80.8 mg/L, approximated to 80 mg/L)
  - IL-6 (> 39.7 pg/mL, approximated to 40 pg/mL)
  - Creatinine (> 1.13 mg/dL, approximated to 1.1 mg/dL)
  - Myoglobin (> 63.34 ng/mL, approximated to 65 ng/mL)
  - TLeuc (> 9570/μL, approximated to 10,000/μL)
  - 25(OH)D (> 38 ng/dL, approximated to 40 ng/dL)
  - ALT (> 42 U/mL, approximated to 40 U/mL)
  - AST (> 48 U/mL, approximated to 50 U/mL)

#### Pulmonary involvement as assessed by chest CT

Pulmonary involvement was based on the chest CT performed 48 h pre- or post-hospitalization. All patients were scanned in the supine position from the lung apex to the diaphragm during end-inspiration using a 64-slice CT scanner. We recorded the presence of typical factors such as ground-glass reticular opacities, with or without consolidations, were quantified, and the pulmonary involvement, in percentages.

For this study, chest CT involvement was classified as:

I- No involvement (no affected area)
II- Mild impairment (affected area up to 24%)
III- Moderate impairment (affected area 25–49%)
IV- Severe impairment (affected area > 50%)

#### Outcomes

The clinical outcomes evaluated were severe respiratory failure with the need for mechanical ventilation (SRF/MV), death during the hospital stay, and up to 30 days after discharge. These outcomes were assessed by daily monitoring of the electronic medical record by a study team member. Vital status was confirmed by each subject’s physician or by the hospital’s medical team.

#### Statistical analyses

The sample size was based on previous literature [14–17] and an estimate of the number of participants admitted to the hospital with a confirmed SARS-CoV-2 infection.

For the analyses, the four participants who remained hospitalized on the final day of the follow-up were included; however, their data were truncated at the end of the study.

To compare groups, the chi-square test and the analysis of variance were used for qualitative and quantitative variables, respectively.

To determine which factors predicted outcomes, we initially evaluated those that showed significance in the correlation test with *p* < 0.09. Then, a logistic regression analysis was performed for each factor in a univariate form; factors that remained significant were then evaluated in an adjusted analysis. In the end, only factors with *p* < 0.05 were considered statistically significant. Statistical software SPSS version 22.0 (SPSS, Inc., Chicago, IL, USA) was used for all analyses. Statistical significance was set at *p* < 0.05.

## Results

The mean age was 72.7 years (± 9.2 years), with 44 (21.9%) aged ≥80 years (Table 1). The vast majority were Caucasian (*n* = 187, 93%) and 127 (63.2%) were men. Our sample was composed of highly educated people (average of 13.9 years of study, 61.5% of whom had more than 13 years of formal education) compared to the average in the population (9.4 years) [18]. At the end of the study, 181 (90%) were discharged, 15 of whom were readmitted within 30 days (Fig. 1).

**Table 1:** Demographic characteristics according to clinical outcomes

Variables (n, %)	Total (*n* = 201)	Mechanical Ventilation (*n* = 34)	No Mechanical Ventilation (*n* = 167)	p	Death (n = 16)	No Death (*n* = 1850)	p
Age (years) (average ± SD)	74.67 ± 9.44	74.0 ± 10.0	72.4 ± 9.0	0.369	80.6 ± 9.1	72.0 ± 8.9	<.001
Aged ≥80 years	464 (21.9)	9 (26.5)	31 (22.9)	0.652	8 (50)	38(20.5)	*0.010*
Gender							
Male	127 (63.2)	21 (61.8)	106 (63.5)	0.847	9 (56.2)	118 (63.8)	0.593
Female	74 (36.8)	13 (38.2)	61 (36,5)		7 (43.8)	67 (36.2)	
Race							
Caucasian	187 (93.5)	31 (91.2)	156 (93.4)	0.335	16 (100)	171 (92.9)	1.00
Afro descendant	7 (3.5)	3 (8.8)	1 (0.6)		0 (0.0)	2 (1.1)	
Asian	6 (3.0)	0 (0.0)	6 (3.6)		0 (0.0)	6 (3.3)	
Marital Status							
No information	15 (7.5)	2 (5.9)	13 (7.8)	*0.047*	0 (0.0)	15 (8.2)	0.226
Not married	6 (3.0)	3 (8.8)	3 (1.8)		0 (0.0)	6 (3.3)	
Married	146 (73.0)	22 (64.7)	124 (74.7)		12 (75)	134 (72.8)	
Divorced	9 (4.5)	0 (0.0)	9 (5.4)		0 (0.0)	9 (4.9)	
Widower	24 (12.0)	7 (20.6)	17 (10.2)		4 (25)	20 (10.9)	
Education (in years) (average ± SD)	13.67 (±5.52)	12.6 (±5.2)	14.0 (±6.1)	0.198	13.2 (±5.5)	13.9 (±6.0)	0.670
Current smoking	11 (5.5)	3 (8.8)	8 (4.8)	0.400	2 (12.4)	9 (4.9)	0.212
Chronic Diseases							
Hypertension	128 (63.7)	26 (76.5)	102 (61.1)	0.118	13 (81.2)	115 (62.2)	0.177
Chronic diseases (≥ 5)	15 (7.5)	3 (8.8)	12 (7.2)	1.0	1 (6.3)	14 (7.6)	1.0
Diabetes mellitus	81 (40.3)	14 (41.2)	67 (40.1)	1.00	9 (56.2)	72 (38.9)	0.196
Heart failure	11 (5.5)	2 (5.9)	9 (5.4)	1.00	2 (12.4)	9 (4.9)	0.212
Previous myocardial infarction	16 (8.0)	4 (11.7)	12 (7.2)	0.482	3 (18.7)	13 (7.0)	0.120
Atrial fibrillation	20 (10.0)	8 (23.5)	12 (7.2)	*0.012*	7 (43.7)	13 (7.0)	*0.001*
History of Stroke	14 (7.0)	3 (8.8)	11 (6.6)	0.709	2 (12.4)	12 (6.5)	0.608
COPD	31 (15.4)	6 (17.6)	25 (15.0)	0.794	4 (25)	27 (14.6|)	0.278
Asthma	14 (7.0)	2 (5.9)	12 (7.2)	1.00	2 (12.4)	12 (6.5)	0.608
Osteoporosis	40 (19.9)	9 (26.5)	49 (29.3)	0.344	4 (25)	36 (19.5)	0.743
Fracture in last 20 years	57 (28.4)	12 (35.3)	45 (26.9)	0.403	4 (25)	53 (28.6)	0.788
Osteoarthritis	57 (28.4)	8 (23.5)	31 (18.6)	0.541	7 (43.7)	50 (27.0)	0.245
Parkinson disease	9 (4.5)	1 (2.9)	8 (4.8)	0.712	0	9 (4.9)	0.621
Alzheimer disease	16 (8.0)	1 (2.9)	15 (9.0)	0.318	2 (12.4)	14 (7.6)	0.621
Chronic kidney disease (non-HD)	8.0 (16)	7 (20.6)	9 (5.4)	*0.012*	4 (25.0)	12 (6.5)	*0.033*
End-Stage Renal Disease (on HD)	5 (2.5)	3 (8.8)	2 (1.2)	*0.035*	0.00	5 (2.7)	1.00
Current cancer	15 (7.5)	2 (5.9)	13 (7.8)	0.753	1 (6.2)	14 (7.6)	1.00
Previous cancer	29 (14.4)	7 (20.6)	22 (13.2)	0.284	5 (31.2)	24 (13.0)	*0.060*
Lung involvement > = 50%	50 (25.3)	19 (55.9)	31 (18.9)	< *0.001*	6 (40)	44 (24)	0.215

Legend: *COPD* Chronic obstructive pulmonary disease, *HD* hemodialysis

Thirty-four (16.9%) participants progressed to respiratory failure with the need of mechanical ventilation, and 16 (8%) died (15 in-hospital and one during the 30-day follow-up after discharge) (Table 2).

**Table 2:** Laboratory results according to clinical outcomes

Variables (n. %)	Total (n = 201)	Mechanical Ventilation (n = 34)	No MV (n = 167)	p	Death (n = 16)	No death (n = 185)	p
***Laboratory tests abnormalities (in quartiles)***							
Higher CRP (mg/L)	51 (25.4)	19 (55.9)	32 (19.2)	*< 0.001*	9 (56.3)	42 (22.7)	*0.006*
Higher IL-6 (pg/mL)	49 (24.4)	15 (44.1)	34 (20.4)	*0.004*	9 (56.3)	40 (21.6)	*0.004*
Higher d-Dimer (ng/mL)	51 (25.4)	18 (52.9)	33 (19.8)	*< 0.001*	10 (62.5)	41 (22.2)	*0.001*
Higher ALT (U/mL)	51 (25.4)	9 (26.5)	42 (25.1)	0.512	3 (18.8)	48 (25.9)	0.384
Higher AST (U/mL)	51 (25.4)	16 (47.1)	35 (21.0)	*0.002*	6 (37.5)	45 (24.3)	0.191
Lower Hemoglobin (g/mL)	51 (25.4)	18 (52.9)	33 (19.8)	*< 0.001*	10 (62.5)	41 (22.2)	*0.001*
Higher Leucocytes (/μL)	49 (24.4)	12 (35.3)	37 (22.2)	*0.082*	7 (43.8)	42 (22.7)	*0.063*
Higher Creatinine (mg/dL)	50 (24.9)	14 (41.2)	36 (21.6)	*0.017*	9 (56.3)	41 (22.2)	*0.005*
Higher Myoglobin (ng/mL)	50 (25.0)	19 (55.9)	31 (18.7)	*< 0.001*	9 (56.3)	41 (22.3)	*0.005*
Lower Vitamin D (ng/dL)	48 (23.9)	45 (26.9)	3 (8.8)	*0.026*	2 (12.5)	46 (24.9)	0.215
Lower Lymphocytes (/μL)	49 (24.4)	17 (50.0)	32 (19.2)	*< 0.001*	6 (37.5)	43 (23.2)	0.165
***Laboratories variables (means ± SD)***							
CRP (mg/L)	54.88 (58.92)	94.97 (68.87)	46.71 (53.32)	< 0.001	98.28 (67.97)	51.12 (56.73)	0.002
IL-6 (pg/mL)	39.50 (60.58)	83.87 (99.40)	30.46 (44.41)	< 0.001	96.58 (85.19)	34.56 (55.59)	0.000
D-Dimer (ng/mL)	886.16 (1002.79)	1426.05 (1432.77)	776.25 (855.34)	0.002	1382.06 (977.41)	843.28 (995.95)	0.039
ALT (U/mL)	39.45 (33.84)	37.88 (27.89)	39.77 (34.99)	0.767	37.00 (33.70)	39.67 (33.94)	0.763
AST (U/mL)	35.20 (23.53)	47.00 (30.85)	32.80 (21.06)	0.001	50.87 (39.25)	33.84 (21.29)	0.005
Hemoglobin (g/mL)	12.77 (1.94)	11.73 (2.16)	12.98 (1.83)	0.001	11.23 (2.18)	12.90 (1.86)	0.001
Leucocytes (/μL)	7012.95 (3410.39)	8552.08 (3246.08)	6699.60 (3366.65)	0.004	9013.12 (3116.15)	6839.97 (3387.12)	0.014
Creatinine (mg/dL)	1.05 (0.63)	1.31 (0.865)	1.00 (0.56)	0.009	1.27 (0.48)	1.03 (0.64)	0.146
Myoglobin (ng/mL)	68.25 (147.88)	163.19 (328.13)	48.81 (51.03)	< 0.001	135.82 (180.03)	62.38 (143.84)	0.057
Vitamin D (ng/dL)	33.01 (15.47)	28.41 (9.98)	33.95 (16.22)	0.057	29.62 (9.89)	33.30 (15.84)	0.362
Lymphocytes (/μL)	1019.56 (599.60)	697.61 (403.83)	1085.10 (612.52)	0.001	863.62 (427.83)	1033.04 (611.20)	0.279

Legend: C-reactive protein (CRP), interleukin 6 (IL-6), alanine transaminase (ALT), aspartate aminotransferase (AST)

### Death

The patients who died were older (> 80 years), had a higher prevalence of chronic atrial fibrillation, CKD, and a tendency to have had a history of cancer. There was no gender predominance, and all were Caucasian. Severe pulmonary involvement at the beginning of the study was not statistically associated with death during hospitalization. The laboratory characteristics of the elderly who died were high levels of CRP, IL-6, D-dimer, leukocytes, and creatinine, and low hemoglobin levels.

**Fig. 2:**
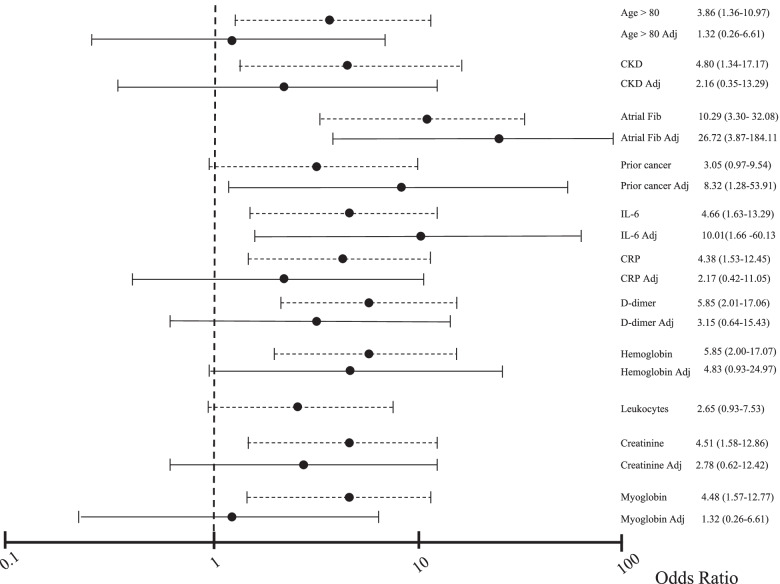
Odds ratio graph for death (non-adjusted and adjusted). Legend: dotted line (−-----): non-adjusted logistic regression analyses; continuous line (  ): adjusted logistic regression analyses for all significant variables. Variables evaluated without adjustment: Age > 80 = individuals aged 80 years or more; CKD = chronic kidney disease; Prior cancer, Atrial Fib = prior atrial fibrillation, IL-6 = interleukin 6 > 40 pg/mL, CRP = C-reactive protein> 80 mg/L; D dimer = d dimer> 1000 ng/ml, Hemoglobin = hemoglobin< 12 g/mL; Leucocytes = leucocytes > 10.000 μL; Creatinine = creatinine > 1.1 mg/dL; Myoglobin = myoglobin> 65 ng/mL). Variables evaluated with adjustment: Age > 80 Adj = individuals aged 80 years or more; CKD Adj = chronic kidney disease; Prior cancer Adj, Atrial Fib Adj = prior atrial fibrillation, IL-6 Adj = interleukin 6 > 40 pg/mL; CRP Adj = C-reactive protein> 80 mg/L; D dimer Adj = d dimer> 1000 ng/ml, Hemoglobin Adj = hemoglobin< 12 g/mL; Creatinine Adj = creatinine > 1.1 mg/dL; Myoglobin Adj = myoglobin> 65 ng/mL)

Most deceased patients had hospital stays beyond 18 days (*n* = 12; 75%; *p* < 0.001), were transferred to the ICU (*n* = 13; 81.3%; p < 0.001), and almost all required mechanical ventilation (*n* = 14, 87.5%; p < 0.001). Higher leucocytes lost statistical significance in the univariate logistic regression analysis, and history of cancer remained borderline (Table 2), while the others remained significant. On adjusted logistic regression analysis, atrial fibrillation, history of cancer, and high IL-6 levels remained significant, while anemia remained borderline (Fig. 2).

### Severe respiratory failure and mechanical ventilation

Elderly patients who progressed to SSRF/MV were characterized by a higher prevalence of chronic atrial fibrillation, chronic kidney disease (non-HD), and end-stage renal disease (on HD). Severe pulmonary involvement and higher levels of CRP, IL-6, D-dimer, AST, creatinine, myoglobin, and lower levels of Tlymp, Hb, and 25(OH)D were associated with respiratory failure (Table 1). 14 (41.2%) died among these patients, and 30 (88.2%) stayed in the hospital for a prolonged period.

**Fig. 3:**
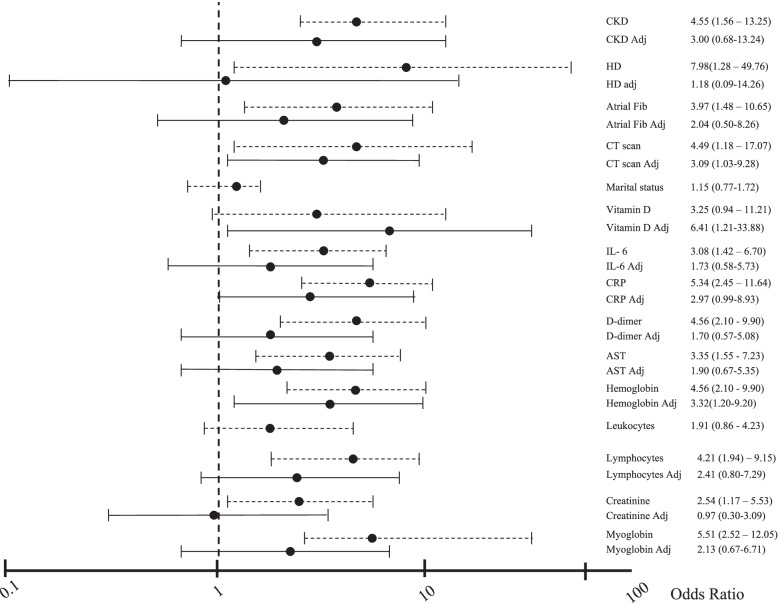
Odds ratio graphs for severe respiratory failure (non-adjusted and adjusted). Legend: dotted line (------): non-adjusted logistic regression analyses. Continuous line (  ): adjusted logistic regression analyses for all significant variables. Variables evaluated without adjustment: Age > 80 = individuals aged 80 years or more; CKD = chronic kidney disease; CT scan = thomographic lung involvement over 50%; Marital status; Atrial Fib = prior atrial fibrillation, Vitamin D = vitamin D < 40 ng/dl; IL-6 = interleukin 6 > 40 pg/mL, CRP = C-reactive protein> 80 mg/L; D dimer = d dimer> 1000 ng/ml, Hemoglobin = hemoglobin< 12 g/mL; Leucocytes = leucocytes > 10.000 μL; Lymphocytes = lymphocytes < 600/μL; AST = aspartate aminotransferase > 50 U/mL; Creatinine = creatinine > 1.1 mg/dL; Myoglobin = myoglobin> 65 ng/mL). Variables evaluated with adjustment: Age > 80 Adj = individuals aged 80 years or more; CKD Adj = chronic kidney disease; CT scan Adj = lung involvement over 50%; Marital status Adj; Atrial Fib Adj = prior atrial fibrillation, Vitamin D Adj = vitamin D < 40 ng/dl; IL-6 Adj = interleukin 6 > 40 pg/mL, CRP Adj = C-reactive protein> 80 mg/L; D dimer Adj = d dimer> 1000 ng/ml, Hemoglobin Adj = hemoglobin< 12 g/mL; AST Adj = aspartate aminotransferase > 50 U/mL; Creatinine Adj = creatinine > 1.1 mg/dL; Myoglobin Adj = myoglobin> 65 ng/mL)

In the analyses with all statistically significant variables in the univariate logistic regression, severe pulmonary involvement, high CRP, low hemoglobin, and vitamin D predicted respiratory failure with the need for mechanical ventilation (Fig. 3).

### Concomitant medications

During hospitalization, 182 (90.5%) participants received oral or intravenous corticosteroids, (1.99%) convalescent plasma, 3 (1.49%) received tocilizumab, 4 (1.99%) received oseltamivir and 1 (0.49%) participant received remdesevir. These medications did not influence the outcomes.

## Discussion

The main contribution of this study was our demonstration that there were significant associations between clinical, radiological, and laboratory parameters with the risk of progression to SRF/MV and death during hospital stays due to COVID-19 in an elderly population.

Anemia as an independent predictor of SRF/MV and mortality remains controversial. A meta-analysis [19] regarding hematologic parameters showed no significant difference between the hemoglobin levels of those who progressed to severe disease or death and patients who did not present these outcomes. Nevertheless, Tao et al. found results similar to ours; they observed that anemia diagnosed in the first 24 h of hospitalization significantly increased the risk of evolution o severe COVID-19 [20]. Wang et al. also found that hemoglobin levels were significantly lower among patients who needed ICU than those who did not [21]. Lower hemoglobin levels have been reported in COVID-19 patients [22, 23] and this may be associated with the intensity of the inflammatory process that interferes with the iron metabolism mediated by the increase in IL-6 and by the direct action of pro-inflammatory cytokines such as interferon-γ, IL-1, IL-33, and tumor necrosis factor (TNF)-α on erythropoiesis, characterized by decreased erythrocyte synthesis [24–26].

When evaluating the outcomes separately, lung involvement above 50%, CRP > 80 mg/L, and vitamin D < 40 ng/mL remained independent risk factors for SRF/MV. The prognostic value of lung involvement evaluated by chest CT scan varied substantially according to the type of population and period of disease [27]. Colombi et al. [28] showed that, in patients in the emergency room, radiological compromise appeared to predict progression to respiratory failure, need for ICU admission, and death, even among those with mild clinical symptoms. Cereser et al. evaluated high-resolution chest CTs of 77 hospitalized patients and observed that, after adjusting for age and comorbidity, severe pulmonary impairment increased the risk for respiratory failure by approximately eight times [29]. Turcato et al. demonstrated an inverse relationship between arterial oxygen pressure and pulmonary impairment, indicating that lung involvement can be a marker of functional impairment resulting from the inflammatory process [30]. Zhang et al. observed a significant difference between the average of inflammatory markers in patients with severe pulmonary involvement compared to those with less radiological involvement [31].

Our findings corroborate these previous findings; the mean of inflammatory markers (CRP and IL 6) in our study was about twice as high among those who progressed to SRF/MV (Table 2). Other factors related to the aging process, such as a decrease in the number of type II pneumocytes, alveolar stiffening, decreased exchange of carbon dioxide and oxygen, lower vital capacity, and respiratory muscle weakness resulting from sarcopenia, may also contribute to early respiratory failure in the elderly [32]. In our view, and that of others, the inflammatory process appears to play a decisive role in the evolution of these patients to SRF/MV and death, even more so among the elderly that might already suffer from Inflammaging [4].

One of the laboratory parameters that surprised us was that serum vitamin D < 40 ng/dl was an independent risk factor for SRF/MV, even after adjusting for all other factors established in the literature [15–17, 26, 28, 30, 31]. In our study, low vitamin D was a significant predictor of SRF/MV but not for death, and this could be due to the participation of vitamin D in the immune system through several pathways. It stimulates macrophages to produce 1,25 (OH)-2-vitamin-D that acts as an anti-inflammatory, inhibiting the activation of B-cells and immunoglobulin synthesis [33], and as an anti-infectious agent by inducing the production of IL-10 in Treg cells. It also promotes Th2 cells that limit the inflammatory process and decreases the production of tumor necrosis factor α [34–36]. It is worth mentioning that the critical inverse relationship between serum vitamin D levels and IL-6 levels is considered one of the principal factors of the inflammatory storm [37]. Another factor related to the lower vitamin D effect can be attributed to its influence on the renin-angiotensin system, and ACE2 receptors found in various organs [38] and decrease with aging [39]. The decrease in ACE2 receptors has been associated with higher mortality by COVID-19, whereas the higher expression, mainly in the lungs, was related to a better outcome and a lower incidence of acute lung injury [40]. Vitamin D increases the expression of ACE2 receptors but also modulates the genetic expression of renin, resulting in a smaller effect of renin on its receptor; that is, there is less chance of pulmonary vasoconstriction and myocardial injury, less renal perfusion, pulmonary edema, and renal and myocardial failure seen in severe COVID-19 [41–44]. The protective effect of vitamin D by the renin-angiotensin system concerning COVID-19 remains unclear. The use of prior vitamin D supplementation was high (56%); however, this fact did not significantly modify outcomes. Nevertheless, the significant differences among quartiles in the percentage of participants using vitamin D demonstrated that the lower risk of poor outcomes observed in the upper quartile might be related to the previous use of vitamin D. Although this may suggest that vitamin D supplementation may act as a protective factor, studies of vitamin D supplementation and its protective effect remain controversial [45–47]. Recently, a study evaluated high doses of vitamin D versus placebo and found no significant changes in the length of hospital stay and mortality from COVID-19 [48]^*.*^; on the other hand, another study found that vitamin D supplementation was associated with a trend toward higher mortality [49], and yet another study found a significant reduction in the need for ICU care [50].

Our study’s overall mortality rate was 8%, with a higher rate in participants aged ≥80 years (17.8%). Bonanad et al. showed a mortality rate in people over 60 years of 12.1, and 29.6% among those over 80 years old with COVID-19 [51]. Contrary to other studies, age above 80 years did not remain a significant factor after adjustment for several variables, suggesting that the clinical vulnerability determined by chronic diseases and the intensity of the inflammatory process may surpass age as a determinant of death. In our study, atrial fibrillation and previous cancer history remained significant after adjusting for inflammatory variables. The influence of these comorbidities with factors independent of death suggests that a phenotype of physical frailty may be present. However, we did not detect a significant correlation between the diagnosis of frailty and death when using the adapted FRAIL scale. This was also noted by Baktashet et al. that showed that frailty, diagnosed by the Rockwood questionnaire [52], did not change the mortality rate or the risk of a more extended hospital stay [16]. Some factors may explain this finding, including the type of instrument used to diagnose frailty, as both the FRAIL [13] and the Rockwood [52] questionnaires are subjective. In our study, the subjectivity bias may have been even greater, since in about 50% of the cases, information was collected from the caregiver, who may under or overestimate the frailty criteria. We opted not to use instruments as the one described by Fried et al. [53] as they involve objective measures of physical performance, and this could pose a risk of COVID-19 transmission to the research team, and the clinical condition of the participant with respiratory difficulty could modify the physical performance tests.

Our study has limitations. The study population may have a selection bias as the refusal by some physicians and participants/family members could be related to the severity of the clinical condition; consequently, we may have underrepresented the number of individuals with a higher chance of death. In our study, it was not possible to distinguish whether the anemia was acute or chronic, since we did not specifically ask about previous anemia nor did we have access to previous laboratory tests. During hospitalization we did not follow D dimer levels of the patients, nor did we evaluate whether the physicians prescribed new AC drugs or changed old ones. This did not enable us to assess whether AC significantly modified the rate of death and respiratory failure. We did not assess respiratory parameters such as respiratory rate, oxygen pressure, and arterial CO_2_ values, which are significant predictors of severe outcomes.

## Conclusions

In hospitalized older adults with COVID-19, tomographic pulmonary involvement > 50%, anemia, vitamin D below 40 ng/mL, and CRP above 80 mg/L are independent factors for progression to severe respiratory failure and the need for mechanical ventilation. The presence of chronic atrial fibrillation, previous cancer, IL-6 > 40 pg/mL, and anemia were independent predictors of death.

### Implications

Studies are still needed to better understand the etiology of anemia in the older adults hospitalized for COVID-19, as well as whether its correction may result in modification of severe outcomes. The identification of the risk factors described above may be of great importance for early adoption of therapies in these patients. Vitamin D supplementation to achieve levels equivalent to or greater than 40 ng/ml may be useful in elderly patients, with the goal of improving immunity and achieving better control of the inflammatory response. However, randomized controlled studies in elderly patients are still needed to confirm this possibility.

## Data Availability

The datasets used and/or analyzed during the current study are available from the corresponding author on reasonable request.

## Abbreviations

25(OH)D: 25-hydroxyvitamin-D
ALT: alanine transaminase
AST: aspartate aminotransferase
CKD: chronic kidney disease (non-hemodialysis)
Creat: creatinine
CRP: C-reactive protein
CT: computed tomography
ESRD: end-stage renal disease (on HD)
HD: hemodialysis
Hb: hemoglobin
ICU: intensive care units
IL: interleukin
LDH: lactate dehydrogenase
Myo: myoglobin
SRF/MV: severe respiratory failure with the need of mechanical ventilation
TLeuc: total leucocytes
TLymp: total lymphocytes
TNF: tumor necrosis factor
